# Erratum

**DOI:** 10.4269/ajtmh.19-0303err

**Published:** 2019-11

In “Case Report: Imported Melioidosis from Goa, India, to Israel, 2018” (https://doi.org/10.4269/ajtmh.19-0303) by Brosh-Nissimov and others, the name of an additional co-author has been inadvertently omitted. Dr. Shay Weiss has been involved in the culturing of the *Burkholderia* isolate for its genetic characterization.

The new list of authors should therefore be Tal Brosh-Nissimov, Daniel Grupel, Shlomi Abuhasira, Hanna Leskes, Ma’ayan Israeli, Shirley Lazar, Uri Elia, Ofir Israeli, Adi Beth-Din, Erez Bar-Haim, Inbar Cohen-Gihon, Anat Zvi, Shay Weiss, Ofer Cohen, and Theodor Chitlaru.

